# The Essential Role of microRNAs in Inflammatory and Autoimmune Skin Diseases—A Review

**DOI:** 10.3390/ijms24119130

**Published:** 2023-05-23

**Authors:** Klaudia Dopytalska, Anna Czaplicka, Elżbieta Szymańska, Irena Walecka

**Affiliations:** 1 Department of Dermatology, Centre of Postgraduate Medical Education, 02-507 Warsaw, Poland; 2 Department of Dermatology, The National Institute of Medicine of the Ministry of the Interior and Administration, 02-507 Warsaw, Poland

**Keywords:** microRNA, skin disease, autoimmune disease, psoriasis, atopic dermatitis, hidradenitis suppurativa, vitiligo, lichen planus, autoimmune blistering diseases

## Abstract

The etiopathogenesis of autoimmune skin diseases is complex and still not fully understood. The role of epigenetic factors is emphasized in the development of such diseases. MicroRNAs (miRNAs), a group of non-coding RNAs (ncRNAs—non-coding RNAs), are one of the important post-transcriptional epigenetic factors. miRNAs have a significant role in the regulation of the immune response by participating in the process of the differentiation and activation of B and T lymphocytes, macrophages, and dendritic cells. Recent advances in research on epigenetic factors have provided new insights into the pathogenesis and potential diagnostic and therapeutic targets of many pathologies. Numerous studies revealed a change in the expression of some microRNAs in inflammatory skin disorders, and the regulation of miRNA expression is a promising therapeutic goal. This review presents the state of the art regarding changes in the expression and role of miRNAs in inflammatory and autoimmune skin diseases, including psoriasis, atopic dermatitis, vitiligo, lichen planus, hidradenitis suppurativa, and autoimmune blistering diseases.

## 1. Introduction

The interaction between external factors and individual genes is important in terms of increased susceptibility to numerous diseases, including skin conditions. Although the etiology of autoimmune skin diseases is not related to the mutation of a single gene, the role of epigenetic factors is emphasized in the pathogenesis of such diseases [1,2]. Individual gene expression is modulated, and the likelihood of disease is increased through the influence of environmental factors. The concept of epigenetics refers to mechanisms that can modulate gene expression and change the phenotype of cells. Epigenetic changes are not related to a change in the DNA sequence, but they involve acquired chromatin structure modifications and/or nucleotide modifications, affecting the activation of transcription factors of selected genes, resulting in the translation of new mRNA [3].

Epigenetic factors may regulate gene expression in mechanisms at the transcriptional (through histone modification, DNA methylation) and post-transcriptional level (microRNAs and long non-coding RNAs—lncRNAs) [3]. MicroRNAs (miRNAs), a group of non-coding RNAs (ncRNAs), are one of the important post-transcriptional epigenetic factors. Regarding the physicochemical properties, ncRNAs are nucleotide molecules that do not dock proteins but may regulate the translation process. Two main groups of ncRNAs are distinguished—small ncRNAs (shorter than 200 nucleotides) and long ncRNAs (longer than 200 nucleotides) [3,4]. MiRNAs are classified as a short ncRNA. It is a single-stranded RNA molecule, consisting of about 20–30 nucleotides, the precursor of which is a short RNA molecule (shRNA). MiRNAs bind to complementary mRNA regions and are involved in translation regulation by inhibiting protein synthesis by enzymatic mRNA degradation or by creating a physical barrier to transcription factors. They are also an important regulator of enzymes controlling epigenetic changes [3,4]. MiRNAs play an important role in the regulation of the immune response by participating in the process of the differentiation and activation of immune system cells—B and T lymphocytes, macrophages, and dendritic cells [5].

Recent advances in research on epigenetics have provided new insights into the pathogenesis and potential diagnostic and therapeutic targets of many pathologies, including skin diseases [1,6].

MiRNA expression patterns are tissue-dependent and relatively stable during various pathological processes, and the regulation of miRNA expression is a promising therapeutic goal [5,6]. Numerous authors highlighted the role of miRNAs in the pathogenesis of autoimmune and inflammatory skin diseases [1,5,7,8]. This study presents the state of the art regarding changes in the expression and role of miRNAs in inflammatory and autoimmune skin diseases.

## 2. Search Strategies

Electronic literature searches were performed in the PubMed and Embase databases. The authors searched papers on PubMed and Web of Science published until March 2023 using the following search terms: (Psoriasis) OR (Atopic dermatitis) OR (Vitiligo) OR (Lichen planus) OR (Hidradenitis suppurativa) OR (Pemphigus) OR (Pemphigoid) OR (Autoimmune blistering diseases) AND (microRNA) OR (miRNA) OR (MiR) as keywords. A number of 918 results were retrieved through database searches. Duplicates and non-English-language articles were excluded. 

Full-text articles were screened independently by two authors (K.D. and A.C.) for the following inclusion criteria: experimental studies characterizing the role of microRNA, pre-clinical studies about microRNA, differences between subjects with autoimmune or inflammatory skin disease and a control group, and studies measuring the activity or expression of a microRNA in autoimmune or inflammatory disease. From 342 potentially appropriate articles that underwent evaluation, 144 full-text articles were found to be suitable for analysis (hypothesis articles, publications with duplicated patients, clinical reports, not microRNA studies, and not autoimmune or inflammatory skin diseases were excluded. All qualified studies examined the role or expression of microRNA in autoimmune and inflammatory skin diseases. Any disagreements between the two researchers were resolved via consensus with a third independent researcher.

## 3. The Role of miRNAs in Inflammatory and Autoimmune Skin Diseases

### 3.1. Psoriasis

Psoriasis is a chronic inflammatory and T-cell-mediated skin disease, in the course of which pro-inflammatory cytokine pathways are activated, leading to excessive proliferation and disruption in keratinocyte differentiation [9]. The pathogenesis of the disease is complex. Its development involves the nuclear factor κ light chain enhancer of activated B cells (NF-kB), whose activation results in increased expression of proinflammatory cytokines, including tumor necrosis factor-α (TNF-α), interferon-γ (IFN-γ), transforming growth factor-β (TGF-β), and interleukins, including interleukin (IL)-1b, IL-17A, IL-17F and IL-22, and IL-23 [9,10,11].

Genetic factors play a significant role in the pathogenesis of the disease. The mode of inheritance is multifactorial, but the major genetic association has been found with the HLA-Cw*06, located within the major histocompatibility complex (MHC) on chromosome 6. Psoriasis has a strong genetic link to the MHC-I antigen presentation pathway. Altered MHC-I peptide presentation to CD8+ T cells could be an important aspect of psoriasis pathogenesis. CD8+ T cells react against melanocytes in the context of HLA-Cw*06 as skin-specific target cells of the psoriatic autoimmune response. HLA-Cw*06 allele is associated with early onset and severe course of psoriasis [12,13]. In addition to genetic predisposition, the pathogenesis of the disease also includes epigenetics modifications and environmental factors, such as stress, certain medications, nicotinism, obesity, and aspects related to the microbiome. Recent advances in research highlighted significant interplay between microbiome and miRNAs expression [14,15]. MiRNAs could play a role in psoriasis by regulation of hyperproliferation, keratinocyte differentiation, apoptosis, and immune activation [12,14].

*miR-31*: miR-31 is a highly overexpressed microRNAs in the blood serum and keratinocytes of psoriasis patients [7,16,17]. An increased concentration of miR-31 results in increasing the activation of NF-κB signaling, which is one of the key signaling pathways in the pathogenesis of psoriasis [17]. miR-31 also reduces the expression of protein phosphatase 6 (ppp6c), which is a component of the cell cycle regulation system. It results in an increase in the number of cells in the S-phase of the cell cycle, leading to epidermal hyperplasia [17]. Increased miR-31 in keratinocytes is responsible for keratinocyte hyperproliferation and acanthosis. Moreover, miR-31 regulates the production of inflammatory mediators, such as INF-α, IL-1, IL-6, IL-17, and IL-22, and stimulates leukocyte chemotaxis [16,18].

Xu et al. [16] also demonstrated that the inhibition of miR-31 in keratinocytes inhibited the NF-kB signaling pathway via inhibiting serine/threonine kinase 40. It resulted in the reduced production of inflammatory chemokines, such as IL-1b, C-X-C Motif Chemokine Ligand 1 (CXCL1), CXCL5, and CXCL8/IL-8 [16]. Research conducted by Wang et al. [19] on the mouse model indicated that the overexpression of miR-31 caused the upregulation of signal transducer and activator of transcription 3 (STAT3), which further brought about the upregulation of p53 and ultimately led to the development of psoriatic skin lesions [19]. Interestingly, in normal keratinocytes, miR-31 stimulated differentiation by affecting the Notch signaling pathway, which is one of the key regulators of epithelial growth and differentiation [20]. Given the impact of miR-31 on aspects of psoriasis pathogenesis, miR-31 suppression may lead to the decreased expression of proinflammatory cytokines and reduce keratinocyte hyperproliferation [16,17,21].

*miR-21*: Similar to miR-31, miR-21 is another well-studied miRNA that is overexpressed in psoriasis. MiR-21 is a pleiotropic regulator of cell biology, with the ability to stimulate cell proliferation, migration, invasion, inflammatory cascade, and inhibition of apoptosis. Elevated miR-21 levels result in the upregulated state of keratinocyte proliferation [22,23,24]. The phenomenon of apoptosis inhibition may be significant in the skin of psoriasis patients, where miR-21 inhibition stimulates the apoptosis of T cells, which may be a promising way to modulate activated T cells in psoriasis [22]. In addition, in a mouse model of the epidermis, ultraviolet B (UVB) phototherapy was shown to increase miR-21 expression, resulting in the downregulation of tumor-suppressor-programmed cell death protein 4 and was mediated by reactive oxygen species and the mitogen-activated protein kinases (MAPK) signaling pathway [25]. A study by Abdallah et al. [26] showed the upregulation of miR-21-3p in an IMQ-induced psoriasiform mouse model. This upregulation was correlated with IL-22 expression and occurred via STAT3 and NF-κB signaling. The primary induction of miR-21-3p by IL-22 would initiate both KC hyperproliferation and an exacerbated inflammatory response, leading to the formation of a positive feedback loop, which may interfere with miR-21-3p transcription. The results of their study confirmed the proliferative potential of miR-21-3p. The study highlighted the role of the miR-21-3p and the IL-22 axis in the pathogenesis of psoriasis [26]. Thus, miR-21 maintains skin inflammation and activation of T cells and stimulates keratinocytes proliferation [22,23,24,25,26].

*miR-146a*: miR-146a regulates inflammation and the proliferation of keratinocytes involved in the pathophysiology of psoriasis. Research showed an increase in the level of miR-146a in psoriatic skin lesions [18,27]. Moreover, a study by Lelal et al. [28] showed that circulating miR-146a serum levels were upregulated in patients with psoriasis, especially in those with active disease [28]. It is suggested that miR-146a is involved in inhibiting the innate immune response. Mir-146a is considered a negative regulator of inflammation and autoimmune response. It acts as a suppressor of mRNA transcripts of proinflammatory transcription factors and their downstream signaling molecule [18,27]. Srivastava et al. [29] demonstrated that reduced miR-146a levels might contribute to an early onset of psoriasis, exacerbation of skin lesions, overexpression of IL-17, epidermal proliferation, and an increase in neutrophil infiltration [29]. In addition, miR-146a may reduce toll-like TLR-dependent epidermal inflammation via IL-1-receptor-associated kinase-1 and TNF-receptor-associated factor 6 pathways. It may result in mediating the IL-17A signaling to NF-κB and the recruitment of inflammatory cells [30,31].

*miR-203*: miR-203 is a skin-specific miRNA that is overexpressed in psoriatic keratinocytes. The upregulation of miR-203 plays a significant role in keratinocyte differentiation and the pathogenesis of psoriasis [32,33]. Xu et al. [32] demonstrated that upregulated IL-17 induced the expression of miR-203, which activated the Janus kinase signaling pathway. This promoted the secretion of VEGF in immortalized nontumorigenic human epidermal cells (HaCaT) [32]. A study by Xia et al. [30] showed that the expression of liver X receptor-α (LXR-α) and peroxisome-proliferator-activated receptor-γ (PPAR-γ) was downregulated in psoriatic lesions and the overexpression of each was sufficient to inhibit keratinocyte proliferation. miR-203 negatively regulated the expression of LXR-α/PPAR-γ through direct targeting, suggesting that the miR-203-LXR-α/PPAR-γ axis was involved in the regulation of the proliferation of keratinocytes and might be a novel target for psoriasis treatment [30]. Due to its role in keratinocytes differentiation and proliferation, mir-203 could be important in pathogenesis of psoriasis [30,32,33].

*miR-155*: Research showed a significant increase in miR-155 expression in psoriatic skin lesions and peripheral blood mononuclear cells patients with psoriasis [34,35,36]. MiR-155 is involved in keratinocyte proliferation and differentiation, apoptotic processes, and immune response regulation [37,38,39,40]. In psoriasis, miR-155 acts through the phosphatase and tension homolog deleted on chromosome 10 (PTEN) signaling pathway. PTEN is downregulated in psoriasis and the loss of PTEN may cause an accumulation of phosphatidylinositol-3, 4, 5-trisphosphate (PIP3), which further increases alpha-serine/threonine-protein kinase (AKT) activity and leads to decreased apoptosis and increased proliferation [39,40]. miR-155 is classified as a proinflammatory miRNA and may stimulate the production of TNF-α. Furthermore, by inhibiting IL-4 expression, it may promote the differentiation of the immune response towards Th1 [21]. In addition, a study by Soonthornchai et al. [41] revealed that both methotrexate and narrow-band ultraviolet B phototherapy (NB-UVB) significantly downregulated miR-155 expression in psoriatic skin lesions [41]. The study also showed that the overexpression of miR-155 in HaCaT led to the suppression of cell apoptosis and induced cell arrest at the G0/G1 phase. miR155 plays a significant role in apoptosis on HaCaT via caspase-3 (CASP3) [41]. Moreover, Liu et al. [39] demonstrated that miR-155 promoted proliferation, migration, inflammatory response, and metabolite levels of human dermal-derived mesenchymal stem cells, which suggests a pathogenic role of miR-155 in metabolic abnormalities in psoriasis [39].

*miR-125b*: The reduction in miR-125b expression in skin lesions and the peripheral blood in patients with psoriasis was confirmed [18,42]. miR-125b was found to be involved in keratinocyte differentiation and proliferation inhibition through its effect on fibroblast growth factor receptor-2 (FGFR2), which is upregulated in psoriatic keratinocytes [42]. Decreased miR-125b expression may result in increased keratinocyte proliferation and upregulated inflammatory cascade by de-repressed mRNA for TNF-α [42,43]. In addition, it was observed that miR-125b levels might increase during UVB therapy for psoriasis [44].

### 3.2. Atopic Dermatitis

The etiopathogenesis of atopic dermatitis (AD) is complex, and it is still unclear whether the initial symptoms of dermatitis are due to a dysfunction of the epidermal barrier, including a deficiency in filaggrin, loricrin, involucrin proteins, and an imbalance between the production of proteases and antiproteases in the stratum spinosum (the “outside-in” hypothesis) or immune dysregulation related to the innate and acquired Th-2 response (the “inside-out” hypothesis) [45]. A combination of multiple mechanisms co-occurring with variable expression of coding and non-coding molecules is reflected in the heterogeneity of the clinical picture and the occurrence of specific “endotypes” in atopic dermatitis [7,46]. miRNA molecules whose potential role in atopic dermatitis was confirmed in research include miR-146a, miR-155, miR-151a, miR-143, miR-124, miR-1294, miR-335, miR-223, miR-24, miR-191, miR-21, and miR-10a.

*miR-146a*: The overexpression of miR146a was found in the skin and blood serum of patients with atopic dermatitis [47,48]. Its role in the pathogenesis may consist in inhibiting the production of proinflammatory cytokines in keratinocytes and fibroblasts. This is conducted by inhibiting various components of the NF-kB pathway, such as caspase-10-recruitment-domain-containing protein 10 (CARD10) or the IL-1-receptor-associated kinase 1 (IRAK1). In addition, it leads to a decrease in the expression of the proinflammatory cytokines CCL5, IL-8, and ubiquitin D by affecting primary human keratinocytes [27,47]. The role of this molecule was partially confirmed by a study in mouse models of atopic dermatitis, in which miR-146a-deficient mice developed a stronger and earlier inflammatory response, characterized by increased cellular infiltration in the skin and increased levels of proinflammatory cytokines [47]. Moreover, miR-146a-/- mice were observed to have impaired IgE production capacity [49,50]. However, this relationship was not confirmed in the serum samples of patients with atopic dermatitis [49]. The main effect of miR-146a seems in AD to be related to suppressing the expression of proinflammatory molecules in epidermal keratinocytes. 

*miR-155*: The role of miR-155 was well described in the immunopathology of atopic dermatitis. In preclinical studies, miR-155-deficient mice showed impaired Th CD8+ response to viral antigens and decreased IL-2 and INF-γ concentrations [51]. As regards AD, miR-155 was found to be produced by CD4+ T cells and dendritic cells. Its expression was positively correlated with the severity of atopic dermatitis, the number of Th17 cells, the expression of IL-17 mRNA, as well as the concentration of IL-17 in the serum [52,53]. miR-155-deficient mice showed impaired Th CD8+ response to viral antigens and decreased IL-2 and INF-γ concentrations [51]. As regards AD, miR-155 was found to be produced by CD4+ T cells and dendritic cells. An increase in miR155 levels maintained chronic skin inflammation through the modulation of CD4+ T cells and dendritic cells and the action on cytotoxic T-lymphocyte-associated antigen 4 (CTLA-4), which is a negative regulator of T lymphocyte activity [54]. AD was also found to be characterized by a negative correlation of miR-155 and suppressor of cytokine signaling 1 (SOCS-1), which stimulates IL-2, IL-3, IL-6 and IFN-γ, and the Janus kinase/signal transducer and activator of transcription (JAK-STAT) pathway [46]. The overexpression of miR-155 was also described in the case of pediatric AD [55]. Thus, the role of miR-155 in the pathogenesis of AD is associated with both the effect on tight junction formation in keratinocytes and the promotion of Th17 differentiation in T cells [51,52,53,54,55,56].

*miR-151a*: Chen et al. [56] demonstrated that the blood cells of patients with atopic dermatitis possessed significant overexpression of miR-151a compared to healthy patients [56]. The role of miR-151a in the pathophysiology of atopic dermatitis may be related to its inhibitory effect on the IL-12 receptor subunit β2, which plays a role in the differentiation of Th1 helper cells [56].

*miR-143*: This molecule probably plays a role in the pathogenesis of atopic dermatitis by acting on IL-13, which reduces the level of expression of filaggrin, involucrin, and locrin via the STAT6 signaling pathway, thus impairing the epidermal barrier, which was confirmed in studies using primary normal human epidermal keratinocytes (NHEKs). MiR-143 binds directly to the 3′-UTR region of the alpha1 receptor for IL-13, leading to a decrease in its expression and, consequently, to a decrease in the production of proinflammatory cytokines induced by IL-13 itself [57,58]. Zeng et al. [57] demonstrated that miR-143 expression was reduced in the skin of patients with atopic dermatitis [57]. Therefore, miR-143 may serve as a potential prophylactic and therapeutic target in the treatment of atopic dermatitis, acting as a suppressor of IL-13-induced dysregulation of skin barrier proteins in epidermal keratinocytes [58].

*miR-124*: miR-124 is another molecule whose expression is reduced in chronic inflammatory lesions in patients with atopic eczema [59]. It is involved in post-transcriptional modifications leading to the inhibition of p65 protein belonging to the NF-kB family of proteins regulating numerous factors involved in the development of the immune and inflammatory response. Moreover, similar to miR-146a, this molecule was found to have a negative influence on the levels of proinflammatory cytokines, such as IL-8, CCL5, and CCL8 [59].

*miR-1294*: Although the role of miR-1294 is more commonly associated with a suppressive effect in some cancers, including esophageal cancer or squamous cell carcinoma of the skin, recent references have shown that it also plays a role in the pathogenesis of atopic dermatitis. In preclinical trials using AD-like skin models, Yan et al. [60] confirmed that the action of miR-1294 through STAT3 to block the NF-kB pathway had an anti-inflammatory effect and inhibited reactive oxygen species (ROS)-dependent response [60].

*miR-335*: The role of miR-335 comprises the direct inhibition of transcription factor SOX6 in the skin of AD patients, thereby promoting keratinocyte differentiation [61]. According to Liew et al. [61], the expression of this miRNA molecule was the most consistently inhibited and practically absent in skin samples in patients with atopic dermatitis compared to healthy people, in whom it was very highly expressed in the suprabasal differentiating layer [61].

*miR-223*: Patients with atopic dermatitis are characterized by a much higher level of miR-223 expression in the plasma, which is associated with platelet activation [62,63]. Moreover, this level was positively correlated with the severity of symptoms in the course of AD and with plasma TARC levels, which is a strong chemoattractant for Th2 helper cells acting through the CC chemokine receptor [63].

*miR-24*, *miR-191*: As regards miR-24 and miR-191, their increased plasma expression was also confirmed in patients with atopic dermatitis and the levels correlated with each other, which may be suggestive of a common trigger mechanism [64]. The level of molecules correlated with the plasma concentration of platelet factor 4 (PF-4) and the plasma concentration of thymus-and-activation-regulated chemokine (TARC), which, as with miR-223, may indicate the role of platelet activation in the pathogenesis of atopic dermatitis. A potential role attributed to miR-24 is related to the inhibition of interferon gamma expression and, indirectly, Th1 responses. In turn, miR-191 was linked to a direct effect on the mRNA of a special AT-rich sequence-binding protein 1 (SATB1), lowering the level of SATB1 protein responsible for promoting the activation of regulatory T lymphocytes [64].

Although the majority of studies on the role of miRNA in the pathophysiology of atopic dermatitis were conducted in the adult population, scientific reports suggested significant differences between the expression of those molecules in the pediatric and adult populations [65,66]. In pediatric atopic dermatitis, the overexpression of miR-203 and miR-483 was reported in the serum of children aged 6 months to 6 years [67]. Nousbeck et al. [68] analyzed changes in the expression of non-coding molecules in infants with moderate and severe AD. As regards miRNAs that were overexpressed, they identified miR-223-3p and miR-143-3p (previously associated with promoting an inflammatory response) and miR-126-5p (associated with Th2 response) in peripheral blood mononuclear cells. miR-451a was described as a molecule with an inhibited expression, both in peripheral blood mononuclear cells and in the serum [68]. Due to its unique expression in different blood compartments of patients with atopic dermatitis, it may be considered as a biomarker in the case of early, non-invasive diagnosis of this dermatosis. In addition to miR-451a, the reduced serum expression of miR-194-5p was also observed in the pediatric population [69].

### 3.3. Vitiligo

Vitiligo is the most common acquired cause of the depigmentation of the skin and mucous membranes. Researchers have not explained the exact pathomechanisms leading to the destruction of melanocytes resulting in the development of discolored foci on the skin. The so-called “convergence theory” postulated the participation of genetic, autoimmune, biochemical, viral, and oxidative stress factors [70,71]. Recent research has provided more scientific evidence for the role of miRNA molecules in the pathogenesis of vitiligo and their relationship with the direct development and progression of the disease. The deregulation of the expression of a variety of miRNA molecules was demonstrated in the skin [72,73,74,75], serum [72,76,77], and peripheral blood mononuclear cells [78,79] in people with vitiligo. Furthermore, a positive correlation was found between the concentration of miRNA-21 and miRNA-125 in the serum of patients and the stage of the disease, which indicates a potential role of these molecules as prognostic factors [72,80]. The role of miRNA in vitiligo has not been fully elucidated. Functionally important miRNA molecules include:

*miR-21-5p*: The miR-21-5p molecule, also known as miR-21, exhibits reduced expression in peripheral blood mononuclear cells of vitiligo patients and may play a protective role in its pathogenesis, affecting the balance between Treg/Teff in the CD4+ cell population [81]. miR-21 reduces the quantity of effector T cells and cytokines associated with their action and promotes the formation of regulatory T cells and Foxp3, which has been confirmed in clinical trials with vitiligo patients. It is due to a direct effect on STAT3, the expression of which is increased in patients with vitiligo [81]. Conversely, research conducted by Aguennouz et al. [80] demonstrated significant overexpression of miR-21 in the serum of patients with vitiligo, which correlated with the Vitiligo Area and Severity Index (VASI) scale [80]. Moreover, it was observed that the expression of proteins involved in melanogenesis, including SOX5, beta-catenin, and CDK2, decreased in melanocyte cultures after 24 h after transfection with miR-21, which emphasized the role of miR-21 as an important regulator of this process [80].

*miR-25*: The expression of miR-25 is increased both in the serum and in the skin of patients with vitiligo. It probably plays a role in the pathogenesis of vitiligo by participating in mechanisms associated with oxidative stress, leading to the dysfunction and destruction of melanocytes. Melanocyte-inducing transcription factor (MITF) is the direct target molecule of miR-25. It is responsible for its harmful effects on melanocytes. It is also the main regulator of survival and functionality of these cells [82]. In addition to direct effects on melanocytes, miR-25 also acts indirectly; i.e., it inhibits the positive, paracrine effect of the secretion of stem cell factor (SCF) and basic fibroblast growth factor (bFGF) from keratinocytes under oxidative stress [72]. Shi et al. [72] also demonstrated that the expression of the miR-25 molecule was increased under oxidative stress both in human melanocytes and keratinocytes via the demethylation of the miR-25 promoter region. Moreover, similar to miR-21, serum concentrations of miR-25 were positively correlated with disease activity. The molecule is a potential target for new antioxidant drugs in vitiligo [72].

*miR-155*: The expression of miR-155 is increased in the skin of people with vitiligo [73]. This molecule plays a role in its pathogenesis not only by influencing melanogenesis-related targets (e.g., inhibiting genes involved in melanocyte differentiation, such as TYRP1, YWHAE, SDCBP, and SOX10 in melanocytes and YWHAE in keratinocytes) but also by modulating interferon signaling. miR-155 is considered a “proinflammatory miRNA”. It inhibits the suppressor of cytokine signaling 1 (SOCS1), leading to the activation of the JAK-STAT pathway with subsequent interferon signaling, as well as other interferon-related genes, such as IFITM1 and IRF1 [73].

*miR-211-5p*: The expression of miR-211-5p is significantly reduced in the skin of people suffering from vitiligo [83]. It is regulated by MITF. MiR-211 promotes pigmentation in melanoblasts and melanocytes by targeting the reduction in TGFβ receptor 2 expression and inhibition of TGFβ-related pathway signaling, which ultimately translates into the downregulation of the expression of two enzymes affecting melanogenesis: tyrosinase and tyrosinase-related protein 1 [84]. Moreover, miR-211 has the ability to interact with other target molecules: nicotinamide adenine dinucleotide (NAD)-dependent deacetylase and sirtuin 1 (SIRT1), which promotes keratinocyte differentiation and protects cells from UVB-mediated DNA damage [83].

*miR-9*: The overexpression of miR-9 was confirmed in the lesional skin of patients with non-segmental vitiligo [74]. E-cadherin, a molecule responsible for the adhesion of keratinocytes and melanocytes, was described as a direct target of miR-9 in patients with ovarian cancer [85] and acute myeloid leukemia [86]. Impaired mechanisms of adhesion between keratinocytes and melanocytes caused by malfunctioning proteins, such as E-cadherin, are closely related to the pathogenesis of vitiligo [71]. The role of miR-9 and its exact effect on E-cadherin was studied during skin repigmentation after UVB phototherapy. Su et al. [87] demonstrated that miR-9 inhibited both E-cadherin and beta 1-integrin in human lesional vitiligo specimens without affecting the levels of laminin, beta-catenin, and collagen IV in the skin of patients with vitiligo [87]. Seemingly, miR-9 also plays a role in regulating cell migration [88,89]. MiR-9 may participate in vitiligo pathogenesis by modulating the expression of oxidative-stress-related genes in melanocytes [87].

*miR-135a*: Similar to miR-211, SIRT1 is the target of miR-135a [90]. Its expression was significantly increased in skin samples in patients with non-segmental vitiligo [74,91]. The confirmed role of SIRT1 is to protect against aging and the development of other stress-related diseases. The overexpression of miR-135a in non-segmental vitiligo lesions leads to the inhibition of SIRT1 and melanocyte destruction [92].

*miR-196a-2*: Similar to miR-211, the rs11614913 miR-196a-2 polymorphism was found to influence the pathogenesis of vitiligo by affecting the expression of TYRP1 and tyrosinase. The rs11614913C allele in miR-196a-2 reduced the expression of the TYRP1 gene. The production of TYRP1 enzyme protein, which is responsible for promoting reactive oxygen species formation, was then decreased in melanocytes. It led to a reduction in the quantity of intracellular reactive oxygen species in melanocytes, thus translating into a reduction in the risk of early apoptosis of human melanocytes and the development of vitiligo [93]. Cui et al. [94] confirmed that the rs11614913C allele in miR-196a-2 strengthened the inhibitory effect on the expression of tyrosinase in melanocytes of vitiligo patients, which also meant a reduction in the formation of reactive oxygen species, the apoptosis index, and the likelihood of developing vitiligo [94].

*miR-377*: miR-377 is overexpressed in the serum of patients with vitiligo, as confirmed by Alhlef et al. [95]. The molecule is responsible for inducing aging in human skin fibroblasts (HSFs) by acting on DNA methyltransferase 1 (DNMT1). It also regulates angiogenesis [96]. In addition, miR-377 was shown to increase oxidative stress in mesangial cells, lead to increased fibronectin accumulation, negatively regulate the expression of SOD1 and SOD2 proteins, and be responsible for increasing the phosphorylation of p38MAPK and the expression of proteins interacting with thioredoxin [95].

*miR-493-3p*: The expression of miR-493-3p is significantly increased in circulating exosomes in patients with segmental vitiligo [97]. Heterogeneous nuclear ribonucleoprotein U (hnRNPU) is an identified target of miR-493-3p. hnRNPU binds to and regulates the main enzyme responsible for the breakdown of dopamine, i.e., catechol-O-methyltransferase (COMT). Recent research has shown that the overexpression of miR-493-3p in keratinocytes increases dopamine concentration, which leads to a significant increase in the production of free oxygen radicals, a decrease in proliferation, as well as an increase in melanocyte apoptosis and melanin synthesis [97].

### 3.4. Lichen Planus

Lichen planus (LP) is a chronic, T-cell-mediated disease of the skin and mucous membranes with a complex pathogenesis. Both genetic and environmental factors are involved in the development of the disease. A subepithelial/subepidermal-band-like lymphocyte infiltrate composed of CD4+ lymphocytes and CD8+ cytotoxic cells was observed in the course of LP [98]. Research revealed a change in the expression of some microRNAs in LP.

*miR-203 and miR-125b*: A study by El-Rifaie et al. [99] showed a decrease both in miR-203 and miR-125b levels in the skin lesions of patients with cutaneous LP [99]. Both miRNAs exert an effect on apoptosis and inflammation. miR-125b is considered a negative regulator of TNF-a, which is involved in inflammatory processes in the skin, including LP. Low levels of miR-125b in LP may enhance the process of apoptosis, which is important in disease progression [99]. The authors suggested that the downregulation of miR-203 and miR-125b might contribute to increased proliferation and decreased differentiation of keratinocytes and enhance apoptosis and recruitment of inflammatory cells [99].

Considerably more authors described the potential role of microRNA in oral LP (OLP). According to research, the expression of miR-155 and miR146a was increased in the mucosal lesions of patients with OLP [100,101,102].

*miR-146a*: A study by Wang et al. [102] showed the upregulation of forkhead box P3 (Foxp3) and miR-146a in OLP tissues and in lipopolysaccharide (LPS)-incubated HaCaT cells. Moreover, Foxp3 inhibition significantly decreased miR-146a expression, ameliorated LPS stimulation by decreased cell proliferation, and decreased apoptosis. miR-146a overexpression upregulated and miR-146a inhibition downregulated the proliferation and apoptosis of LPS-incubated HaCaT cells. Wang et al. suggested that miR-146a could regulate the progression of OLP [102].

*miR-155*: miR-155 may also play an important role in the pathogenesis of OLP. miR-155 was found to upregulate proinflammatory cytokines, such as INF-γ, TNF-α, TGF-β1, and IL-1β [103]. A study by Hu et al. [103] revealed a positive miR-155-IFN-γ feedback loop in erosive OLP CD4+ T cells, which might contribute to the Th1-dominated immune response [104]. MicroRNAs whose expression is downregulated in OLP include miR-214, miR-27a/b, and miR-26a/b [103].

### 3.5. Hidradenitis Suppurativa

Hidradenitis suppurativa (HS) is a chronic inflammatory disease presenting with occlusive disorders affecting the pilosebaceous unit, together with the accompanying immune response of the body. The pathogenesis of this dermatosis is complex and not fully elucidated, with some role being attributed to obesity, diet, nicotinism, mechanical injuries, and hormonal factors [105]. Genetic factors also play an important role in the onset of the disease. It is confirmed that 30–40% of HS patients have a positive family history of this condition [106]. Moreover, genetic variation in the γ-secretase complex (GNS) has been shown in patients with familial HS [107]. Potential new targets for treatment and biomarkers of the disease may include miRNAs molecules and their target molecules, showing both pro- and anti-inflammatory activity [108]. MiRNAs have achieved a GRADE (Grading of Recommendations, Assessment, Development, and Evaluation) rating of moderate susceptibility biomarkers [109]. The role of miRNA in the pathogenesis of HS was supported by the research of Hessam et al. [110]. They demonstrated a significant reduction in the expression of key regulators of miRNA maturation in the skin lesions of HS patients [110]. Subsequently, they proved that the main mechanisms responsible for the formation and functioning of miRNA in patients with HS were characterized by significant dysregulation. Those mechanisms included the following proteins: transactivation-responsive RNA-binding protein-1 (TRBP1), TRBP2, protein activator (PACT) of the interferon-induced protein kinase R, argonaute RISC catalytic component 1 (AGO1) and component 2 (AGO2), metadherin, and staphylococcal nuclease and Tudor-domain-containing 1 (SND1) [111]. Moreover, research showed that the miRNA molecules whose overexpression was identified in the skin of people with hidradenitis suppurativa compared to healthy people were miR-146a-5p, miR-155-5p, miR-223-5p, miR-31-5p, and miR-21-5p [108]. As regards research on miRNA expression in peripheral blood leukocytes in HS patients, increased expression levels were identified for miR-338-5p [112]. Conversely, the expression of miR-24-1-5p, miR26a-5p, miR-206, miR338-3p, and miR146a-5p was reduced in peripheral blood leukocytes [112]. miRNA-125b-5p is a molecule with a reduced expression in the lesional skin compared to apparently healthy skin from the area of inflammatory skin lesions [108].

*miR146a-5p*: De Felice [112] and Hessama et al. [108] suggested that miR146a-5p exhibited variable expression in HS patients. Its overexpression was found in skin samples, while, in peripheral blood leukocytes, its level of expression was significantly reduced compared to the healthy population [108,112]. The dysregulation of this miRNA was also described in atopic dermatitis. As described earlier (AD section), its mechanism of action was found to be associated with the impact on TRAF6/IRAK-1 and the inhibition of TNF-alpha production.

*miR155-5p*: The expression of miR155-5p is significantly increased both in the tissue area affected by inflammation in the course of HS and in the area of apparently healthy skin near the lesions. Its role is to regulate critical immune mechanisms, as described in the case of rheumatoid arthritis and atopic dermatitis. The overexpression of miR-155-5p in samples from patients with rheumatoid arthritis resulted in increased production of proinflammatory cytokines, such as IL-6, IL-1b, IL-8, and TNF-alpha, through negative effects on Src-homology-2-containing inositol phosphatase 1 (SHIP-1) [113]. Both the excessive expression of TNF alpha [114,115] and increased infiltration with Th17 cells in the area of affected skin and lesions were described as pathogenetic mechanisms in hidradenitis suppurativa [116,117]. Such observations may suggest that miR-155-5p dysregulation in HS patients might be responsible for the development of the initial stages of the inflammatory process in the lesional area of the skin [108].

*miR-125b*: miRNA-125b is able to regulate the differentiation and proliferation of keratinocytes through its inhibitory effect on fibroblast growth factor receptor 2 (FGFR2). In the study with HS patients, Hessam et al. [108] proved that miRNA-125b also directly inhibits TNF-alpha [108]. Interestingly, it was shown in the mouse model that the inhibitory effect on its expression was exerted by stimulation with lipopolysaccharides followed by the activation of the NF-kB pathway, mediated by toll-like receptors (TLR) [118]. The mechanism of the activation of this pathway through bacterial lipopolysaccharides located in the deep layers of the skin during processes occurring in the follicle–hair–sebum unit in HS is one of the elements of the immune response occurring in hidradenitis suppurativa [118].

*miR-31-5p*: The miR-31-5p molecule is responsible for regulating the expression of cytokines and chemokines in keratinocytes by blocking serine/threonine kinase 40 (STK40), which is one of the negative regulators of the NF-kB signaling pathway. Blocking miR-31-5p results in a reduction in the production of proinflammatory mediators, such as IL-1b, and a reduction in leukocyte chemotaxis to the skin [108].

*miR-21-5p*: Similar to psoriasis studies, miR-21-5p overexpression was found in the skin lesions of people with hidradenitis suppurativa [22,108]. Meisgen et al. [22] conducted a study on the role of miR-21 in psoriasis and demonstrated that miR-21 overexpression was associated with the activation of T cells, and the role of miR-21 might be associated with the inhibition of their apoptosis, translating into the maintenance of inflammation [22]. Research using mouse models showed that the inhibition of miR-21 resulted in the decreased expression of Th17-related cytokines, such as IL-17A, IL-17F, IL-21, IL-22, and TNF-alpha [119,120].

*miR-223*: The MEF2 transcription factor is the main target of miR-223. It promotes the proliferation of the myeloid line. The activity of miR-223 was found to have a negative effect on the proliferation of progenitors and the differentiation and activation of granulocytes, as proven in studies using mouse models [121]. Interestingly, it was experimentally shown that granulocytes deficient in miR-223 activity were hypersensitive to stimulation and showed increased antifungal activity [121]. The overexpression of miR-223 in skin lesions in the course of hidradenitis suppurativa may be explained by a significant accumulation of neutrophils in the tissue affected by the inflammatory process in this dermatosis [122].

### 3.6. Autoimmune Blistering Diseases

#### 3.6.1. Pemphigus

Pemphigus comprises a group of autoimmune blistering diseases, characterized by the occurrence of acantholysis, resulting in the formation of intraepithelial blisters in the skin and mucous membranes. Antibodies against desmogleins, glycoproteins involved in the formation of desmosomes, play a dominant role in the pathogenesis of pemphigus vulgaris and pemphigus foliaceus. An increasing number of authors described the role of miRNA in pemphigus and sought potential options in predicting its course and targets for new drugs [123,124]. The overexpression of molecules such as miR-338-3p, miR-424-5p, miR-584-5p, miR-125b-5p, miR-155-5p, miR-181a-5p, miR181b-5p, and miR-326 was described in pemphigus vulgaris [125]. The most important molecules from the group include:

*miR-338-3p*: It is one of the miRNA molecules characterized by the greatest overexpression in peripheral blood mononuclear cells in patients with pemphigus compared to the control group [126]. Its potential target genes include ring finger protein 114 (RNF114), tumor necrosis factor receptor type 1 (TNFR1)-associated death domain protein (TRADD), and runt-related transcription factor 1 (RUNX1) [126,127,128]. The first one, RNF114, is a protein involved in regulating the activity of NFkB (nuclear factor kappa light chain enhancer of activated B cells), responsible for inducing the production of proinflammatory cytokines. miR-338-3p plays a role in the negative regulator of the NFkB transcription pathway via stabilizing the A20 and IkB*α* inhibitors. Furthermore, RNF114 is a negative regulator of T cell activation involved in the mechanisms responsible for T cell apoptosis, which is supported by a significant increase in CD69 and CD25 expression after silencing its action [129]. TRADD, the second target gene for miR-338-3p, plays a key role in signal transmission via TNFR1, which, depending on the interaction, may promote proliferation or apoptosis and act on the pathway of NFkB and MAP kinase, e.g., JNK and p38 [130]. As regards RUNX1, the overexpression of miR-338-3p leads to a reduction in the expression of RUNX1 and its dependent transcription factor, FOXP3, in CD4+ cells, which contributes to the dysfunction of regulatory T cells observed in the course of pemphigus [128].

Notably, previously published papers described a positive correlation between the concentration of miR-338-3p and the concentration of antibodies against desmoglein 3, which also correlated with the severity of the disease [127,128]. Additionally, miR-338-3p levels declined significantly after effective treatment, as opposed to the levels of anti-Dsg antibodies, which were significantly reduced only after 6 weeks of effective treatment [127]. These findings suggest the potential role of miR-338-3p as a prognostic factor with its possible use in the diagnosis of pemphigus and the assessment of the severity of the course of this dermatosis.

*miR-424-5p*: The overexpression of this miRNA was confirmed in peripheral blood mononuclear cells in patients with active lesions in the course of pemphigus [131]. The potential pathogenetic effect of miR-424-5p is related to the MAPK signaling pathway, which is dysregulated in the event of changes in the course of pemphigus. p38 MAPK plays a significant role in regulating autoimmune response, cell survival, cell proliferation, differentiation, and apoptosis [132]. Moreover, Chernyavsky et al. [133] described the relationship between the activation of p38 MAPK and the breakdown of the cytoskeleton, its desmosomes, and the final apoptosis of keratinocytes [133]. Pemphigus-specific antibodies are characterized by the ability to undergo such an activation in human keratinocytes [134]. Additionally, the key role of this signaling pathway in the pathogenesis of pemphigus is emphasized by the fact that blocking the activity of p38 MAPK protects against antibody-induced reorganization of the cytoskeleton and the phosphorylation of heat shock protein 27 (HSP27), which plays a role in the formation of the cytoskeleton regarding actin filaments and keratin intermediate filaments [134]. It was assumed that miR-424-5p affected the phosphorylation of HSP27 through an additional effect on increasing the expression of serine/threonine kinase MAPKAPK3 and the CDC25B phosphatase family [135].

*miR-584-5p*: The SRC homology 3 (SH3) and tetratricopeptide repeats 2 (SH3TC2) domains were identified as the target genes for miR-584-5p [136]. He et al. [125] described the overexpression of this miRNA in the serum of patients with pemphigus vulgaris [125]. However, in the case of pemphigus foliaceus, Cipolla et al. [136] reported the occurrence of one of the polymorphisms of the 3′UTR KLRG1 region (killer cell lectin-like subfamily G member 1 receptor), called the rs1805672 G allele, which disrupted the miR-548-5p binding and increased the expression of the KLRG1 gene [136]. As regards active or severe lesions in the course of pemphigus foliaceus, the miRNA concentration of KLRG1 was significantly higher [137]. E-cadherin, one of the main adherens junctions responsible for intercellular adhesion, is the main ligand for KLRG1. The interaction between KLRG1 and E-cadherin was found to lead to changes in cytokine production: decreased production of proinflammatory cytokines and increased production of anti-inflammatory cytokines. It was also responsible for KLRG1-induced phosphorylation of cadherin and its downregulation, resulting in a reduction in cell adhesion [138,139]. Moreover, this interaction, by influencing the activation of various signaling pathways in keratinocytes, led to “apoptolysis”, which is one of pathogenetic mechanisms described in the case of pemphigus [140]. Additionally, the role of interactions between E-cadherin/KLRG1/miR-584-5p and E-cadherin itself in the development of vesicular lesions is emphasized by the fact that 100% of patients with pemphigus foliaceus were found to have anti-E-cadherin antibodies [141].

*miR-326*: This molecule is overexpressed in the serum of patients with pemphigus vulgaris. Furthermore, a statistically significant positive correlation was observed between mild and moderate severity in skin lesions in pemphigus vulgaris [125]. The exact role that miR-326 plays in the pathogenesis of pemphigus and blistering diseases has not been elucidated.

#### 3.6.2. Bullous Pemphigoid

Bullous pemphigoid is an autoimmune blistering disease characterized by the formation of tense subepidermal blisters in the skin and mucous membranes, accompanied by persistent itching [142]. Its pathogenesis includes antibodies against two structures involved in the formation of hemidesmosomes: basal cell membrane adhesion structure 180 (BP180) and 230 (BP230). As a result of their action, the complement components are activated, granulocytes are recruited, proteolytic enzymes are released, and, finally, a blister is formed at the dermal–epidermal border. The role in the pathogenesis, as well as the diagnosis and prognosis in the course of pemphigoid, is also sought in small non-coding miRNA molecules [142]. Compared to healthy subjects, serum concentrations of miR-1291, miR-27a-5p, and miR-423-5p were increased in patients with bullous pemphigoid [143]. Of these, miR-1291 was considered a potential serous biomarker of bullous pemphigoid as its concentration was the only one that decreased after effective treatment. Moreover, Qiu et al. [143] also described a statistically significant positive correlation between serum concentrations of miR-1291 and Th2 chemokine—CCL17 and anti-BP180. Potential target genes for miR-1291 include signal transducer and activator of transcription 6 (STAT6) and IL-13 [143], which are important components of the Janus kinase/signal transducers and activators of transcription (JAK/STAT) signaling pathway, which plays a role in controlling processes such as cell growth, cell differentiation, and apoptosis [144]. The concentration of STAT6, one of the target genes for miR-1291, was significantly increased in the skin lesion samples of patients with bullous pemphigoid. However, it was not increased in skin samples from the area of skin lesions and from the healthy skin. The JAK-STAT signaling pathway was dysregulated in bullous pemphigoid, which might be due to the activation of various cytokines and the formation of neutrophil and eosinophil infiltration [145].

## 4. Conclusions

Multiple studies have identified aberrant expression and function of miRNAs associated with pathological processes, development, maintenance, or progression of skin diseases, including psoriasis, atopic dermatitis, vitiligo, hidradenitis suppurativa, lichen planus, and autoimmune blistering diseases (Table 1).

Selected miRNAs could be involved in the differentiation and activation of B and T lymphocytes, macrophages, and dendritic cells and apoptosis, proliferation, and differentiation of keratinocytes, which are significant processes in the pathogenesis of autoimmune skin disorders. Recent evidence has provided new insight into specific pathways of miRNA involved in such dermatoses. The regulation of miRNA expression could be a diagnostic or therapeutic goal, but further investigations of miRNA-associated regulatory mechanisms are needed.

## Figures and Tables

**Table 1:** The most common aberrant expressed miRNAs in autoimmune and inflammatory skin diseases and their potential function.

miRNA	Level	Diseases	Sample	Potential Effects	References
miR-31	↑	psoriasis	skin lesions, serum	enhances the production of inflammatory mediators and leukocyte chemotaxis to the skin, hyperproliferation of keratinocytes, acanthosis	[7,16,17,18]
	↑	hidradenitis suppurativa	skin lesions		[108]
miR-21	↑	psoriasis	skin lesions	maintains skin inflammation, activation of T cells, stimulates keratinocyte proliferation, inflammatory cascade, and inhibition of apoptosis	[22,23,24]
	↑	hidradenitis suppurativa	skin lesions		[108]
	↓	vitiligo	peripheral blood mononuclear cells	protects melanocytes via targeting STAT3 and modulating Treg/Teff balance to alleviate vitiligo	[81]
miR-146a	↑	psoriasis	skin lesions	regulates inflammation and the proliferation of keratinocytes, inhibiting the innate immune response, controls nuclear-factor-kappa-B-dependent inflammatory responses in keratinocytes and chronic skin inflammation in AD	[18,27,28]
	↑	atopic dermatitis	skin lesions, serum		[47,48]
	↑	oral lichen planus	mucosal lesions		[102]
	↑	hidradenitis suppurativa	skin lesions	effects on TRAF6/IRAK-1 and inhibition of TNF-alpha production	[108,112]
miR-155	↑	psoriasis	skin lesions, peripheral blood mononuclear cells	modulates the differentiation and function of Th17 cells, regulates keratinocyte proliferation and differentiation, apoptotic processes, inflammatory response (overexpression of TNF-alpha, increasing infiltration with Th17 cells)	[37,38,39,40]
	↑	atopic dermatitis	skin lesions, serum		[51,53]
	↑	oral lichen planus	mucosal lesions		[103]
	↑	hidradenitis suppurativa	skin lesions		[114,115]
	↑	vitiligo	skin lesions	inhibits genes involved in melanocyte differentiation, activation of JAK-STAT	[73]
	↑	pemphigus	skin lesions	unknown	[125]
miR-203	↑	psoriasis	skin lesions	plays a significant role in keratinocyte differentiation and proliferation	[32,33]
		children atopic dermatitis	serum		[67]
		cutaneous lichen planus	skin lesions		[99]
miR-125b	↓	psoriasis	skin lesions	inhibits TNF-alpha, regulating the differentiation and proliferation of keratinocytes through its inhibitory effect on the fibroblast growth factor receptor 2 (FGFR2)	[18,42]
	↓	hidradenitis suppurativa	skin lesions		[108,118]
miR-24	↑	atopic dermatitis	serum	inhibits interferon gamma expression	[64]
	↓	hidradenitis suppurativa	peripheral blood leukocytes		[112]
miR-223	↑	atopic dermatitis	serum	relates to platelet activation and plasma TARC levels	[62,63]
	↑	hidradenitis suppurativa	skin lesions	negatively affects the proliferation of progenitors and the differentiation and activation of granulocytes	[121,122]
miR-338-3p	↓	hidradenitis suppurativa	peripheral blood leukocytes	-	[112]
	↑	pemphigus vulgaris	peripheral blood mononuclear cells	has an impact on dysfunction of Treg	[126]

Arrows indicate significant up- or downregulation of microRNA expression (↑—upregulated; ↓—downregulated).

## Data Availability

Not applicable.
